# Polypharmacy in Older Adults: The Role of the Advanced Practitioner in Oncology

**Published:** 2013-03-01

**Authors:** Diane G. Cope

As Americans age, health-care providers will be faced with new and challenging issues related to the care of older adults. One critical issue that is unique to the aging population is polypharmacy. Older adults are frequently prescribed multidrug therapy to treat a plethora of chronic illnesses. On average, individuals aged 70 years and older have 3 or more comorbidities (Extermann, 2007; Fortin, Bravo, Hudon, Vanasse, & Lapointe, 2005). In the United States, approximately 60% of older adults take 5 medications and 20% take 10 or more medications daily (Rocchiccioli, Sanford, & Caplinger, 2007; Slone Epidemiology Center at Boston University, 2006). In addition, older adults consume 40% to 50% of all over-the-counter medications (Budnitz et al., 2006).

Polypharmacy increases the risk of adverse drug reactions (ADRs) in the older adult as a result of overmedication, drug interactions, medication errors, or noncompliance, often leading to increased morbidity (Corsonello et al., 2009; Nixdorff et al., 2008).

Cancer-related medications can add to the complexity of polypharmacy related not only to cancer therapy but also to the multitude of supportive medications used by the cancer patient. Furthermore, other challenges in medication management are age-related changes and pharmacotherapeutics. The advanced practitioner (AP) in oncology is in a key position to assess and manage the older adult’s pharmaceutical agents and maximize drug therapy. This article will define polypharmacy and discuss inappropriate agents for older adults, drug interactions, pharmacotherapeutics, age-related changes, pharmacodynamics, and the role of APs in oncology in ensuring drug safety and reducing adverse drug reactions in the older adult with cancer.

## Definitions of Polypharmacy

Several definitions exist for polypharmacy. Initially, polypharmacy was defined as the use of multiple medications, also termed *polymedicine* (Pham & Dickman, 2007; Rocchiccioli, Sanford, & Caplinger, 2007; Stewart, 1990), although no specific number of drugs has been identified in the literature (Planton & Edlund, 2010). The definition of polypharmacy has since been expanded beyond the number of medications and now also includes the use of potentially inappropriate medications (PIMs) and drug interactions (Maggiore, Gross, & Hurria, 2010; Planton & Edlund, 2010; Rocchiccioli, Sanford, & Caplinger, 2007) that can lead to adverse drug reactions in the older adult. Factors other than multiple medications that can enhance polypharmacy in the older adult patient include multiple prescribers and pharmacies, medications taken without clear clinical indications, and duplicate medications.

## Potentially Inappropriate Medications

Two tools that have been frequently used to evaluate potentially inappropriate medication use in older adults have been the Beers criteria and the Medication Appropriateness Index (MAI) (Maggiore, Gross, & Hurria, 2010). The Beers criteria, based upon expert consensus, consist of a list of medications thought to be inappropriate for use in older adults and are divided into two sections: (1) medications or medication classes that should generally be avoided in persons 65 years or older because they are either ineffective or they pose unnecessarily high risk for older persons and a safer alternative is available and (2) medications that should not be used in older persons known to have specific medical conditions (American Geriatrics Society 2012 Beers Criteria Update Expert Panel, 2012; Fick et al., 2003).

The MAI assesses the degree of appropriateness to a medication using evaluation criteria and a three-point Likert scale (Hanlon et al., 1992). The evaluation criteria include indication, effectiveness, dosage, directions, drug-drug interaction, drug-disease interaction, duplication, duration, and comparative cost.

Another list of high-risk medications available to the health-care provider has been developed by the National Committee for Quality Assurance. The Healthcare Effectiveness and Data Information Set (HEDIS) is a program designed to identify standards of care for clinical measures. The HEDIS recently added a list of "Drugs to be Avoided in the Elderly" that is similar to but not as extensive as the Beers criteria (National Committee on Quality Assurance, 2007). Smartphone users can scan the barcode below to access this list; other readers can visit http://www.ncqa.org/portals/0/newsroom/2007/Drugs_Avoided_Elderly.pdf

## Drug Interactions

A drug-drug interaction is defined as an increase or decrease in the clinical effect of a given drug due to interaction of another drug (Scripture & Figg, 2006). Drug interactions can occur between drugs, and between drugs and food, herbs, or tobacco. Older adults are at high risk for drug interactions due to the incidence of increased comorbidities, multiple medication use, altered nutritional status, age-related physiologic and cognitive changes, and alterations in pharmacokinetics.

Approximately one-third of ambulatory cancer patients are at risk for drug-drug interactions because of the use of multiple medications to treat comorbidities, cancer, and treatment-related toxicities (Riechelmann & Del Giglio, 2009). Pharmacokinetic changes may also occur with impaired absorption due to mucositis and malnutrition and impaired distribution and excretion due to fluid imbalances and organ dysfunction. A summary of drug-drug interactions seen with chemotherapy agents, supportive care agents, and other drugs is presented in Table 1.

**Table 1:**
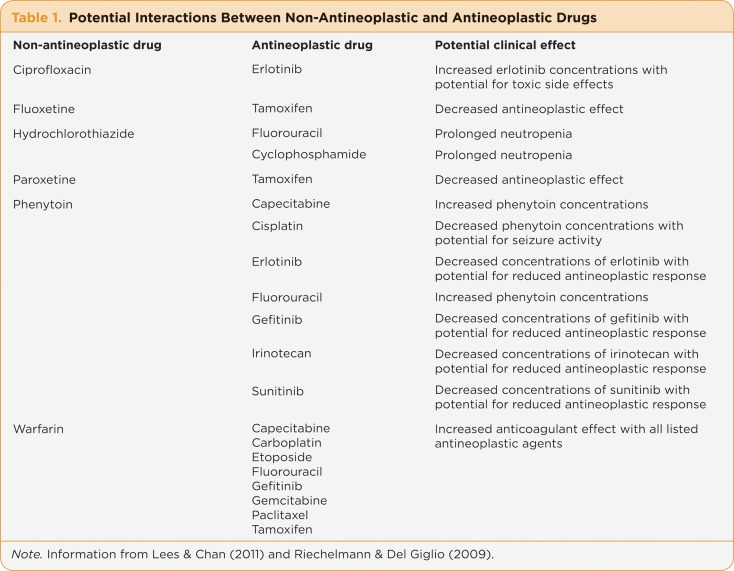
Table 1.Potential Interactions Between Non-Antineoplastic and Antineoplastic Drugs

## Pharmacokinetics and Age-Related Changes

Pharmacokinetics of drug therapy is an important issue in the care of the older adult because of the many age-related changes occurring in this age group (He, Clarke, & McLachlan, 2011; Hurria & Lichtman, 2007). Pharmacokinetics is the physiologic effects of drugs within the body. The four major processes are absorption, distribution, metabolism, and elimination. Age-related changes can significantly impact these processes and alter the expected outcomes of therapy (Table 2).

**Table 2:**
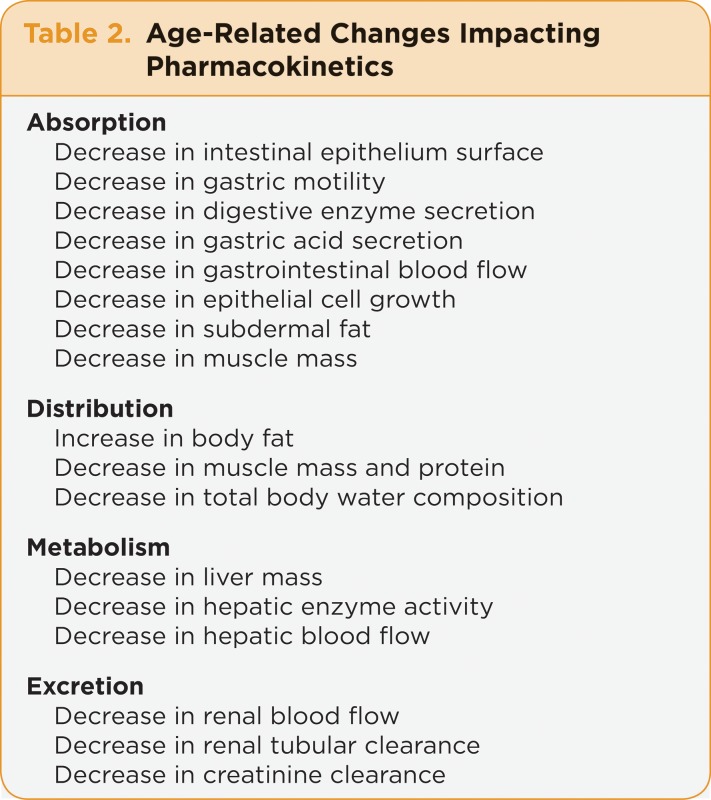
Table 2. Age-Related Changes Impacting Pharmacokinetics

## Absorption

Age-related changes that can occur in the gastrointestinal tract can alter the drug absorption process for oral medications (Schwartz, 2006). A decrease in the intestinal epithelium surface can decrease the area of drug absorption and may increase the length of time medications remain in the tract. A decrease in gastric motility can decrease or increase drug absorption, depending on the medication. A decrease in secretion of digestive enzymes and gastric acid can alter drug disintegration and dissolution and decrease drug absorption. A decrease in gastrointestinal blood flow can decrease drug absorption through the gut wall (He, Clarke, & McLachlan, 2011; Schwartz, 2006; Zurakowski, 2009).

Transdermal medications may also be altered due to age-related skin changes. With age, epidermal cell growth slows, causing thinning of the skin. The skin becomes dry with less subdermal fat (Zurakowski, 2009). Drug absorption of topical medications can be at a different rate or amount when these changes are present in the older adult.

Decreases in muscle mass occur during aging and can alter intramuscular drug absorption. Intramuscular injections may be absorbed faster; however, decreases in absorption may also be seen because of the decreased peripheral blood flow (Zurakowski, 2009).

## Distribution

Drug distribution is dependent upon fat, protein, and water content in the body. Age-related changes result in an increase in body fat and a decrease in muscle mass and protein, and total body water composition (He, Clarke, & McLachlan, 2011; Schwartz, 2006; Zurakowski, 2009). Drugs that are fat-soluble may remain longer in the body and increase the risk of overdose. Lower levels of protein can cause higher serum concentrations of protein-binding drugs such as warfarin. Water-soluble drugs can also be more active as a result of lower body water composition.

## Metabolism

With aging, liver mass, hepatic enzyme activity, and hepatic blood flow are decreased, resulting in alterations in drug metabolism in the liver. These changes may result in alterations in drug metabolism to an inactive form, and therefore there can be longer exposure to an active drug, producing adverse drug effects. Drugs metabolized in the liver to an active form may have less therapeutic effect or a delay in onset of action (Hurria & Lichtman, 2007; Schwartz, 2006).

## Excretion

Age-related changes in renal function have a significant impact on drug clearance. Renal changes in the older adult include reduction in renal blood flow, decrease in renal tubular clearance, and reduction in creatinine clearance (He, Clarke, & McLachlan, 2011; Lacasse, 2011). Older adults with decreased renal function as well as decreased drug clearance are at risk for increased toxicities often associated with drug therapy.

## Pharmacodynamics

Pharmacodynamics is the pharmacologic activity of a drug, defined as the drug concentration at the target organ (Schwartz, 2006). Alterations in pharmacodynamics and end-organ response are affected by age-related changes in effector system function, organ function, and impaired homeostatic control or from multiple concomitant pathophysiologic changes (He, Clarke, & McLachlan, 2011). For the older adult with cancer, efficacy and toxicity of chemotherapy agents may be affected with increased risk of short- and long-term complications such as myelosuppression, mucositis, cardiomyopathy, and peripheral neuropathy.

## The Role of the Advanced Practitioner in Oncology

The oncology AP can significantly affect the care of the older adult with cancer in providing optimal pharmaceutical care and drug safety. The AP plays a key role in monitoring symptom management of oncologic therapy and can prevent adverse drug reactions through ongoing assessment and close follow-up.

Although a comprehensive medicine evaluation should be performed with all older adults, certain key characteristics can place some individuals at higher risk for medication problems (Planton & Edlund, 2010). These characteristics include age ≥ 85, renal insufficiency, low body weight, 6 or more comorbidities, more than 12 dosages of medications per day, more than 9 different medications, and a history of adverse drug reactions.

Several tools are also available to systematically assess medication use. These tools include START (Screening Tool to Alert Doctors to the Right Treatment) and STOPP (Screening Tool of Older Person’s Potentially Inappropriate Prescriptions; Lam & Cheung, 2012) and ARMOR (Assess, Review, Minimize, Optimize, Reassess) (Haque, 2009). The START tool is organized by organ systems and attempts to prevent omission of appropriate medications. The STOPP tool is a useful guide to identify potentially inappropriate medications. The ARMOR tool is a systematic approach to evaluate medications, taking into account functional status and altered physiologic states. This tool emphasizes quality of life and maintenance of functional status in making decisions on medication use, with a consideration of a medication’s impact on bowel and bladder function and appetite.

The AP can also develop an individualized clinical strategy to evaluate medication use in the older adult. Websites are available for additional resources in assessing medications in the older adult and are listed in Table 3. Key components of an assessment should include the following:

1. Review of the patient’s complete medication list including prescription, over-the-counter medications, herbs, and supplements
2. Evaluation of each medication’s indications, benefits, and side effects
3. Review of the Beers criteria
4. Review of potential drug interactions
5. Cognitive function
6. Assessment of activities of daily living
7. Assessment of nutritional status
8. Assessment of hepatic and renal function
9. Financial resources and prescription coverage
10. Social support resources

**Table 3:**
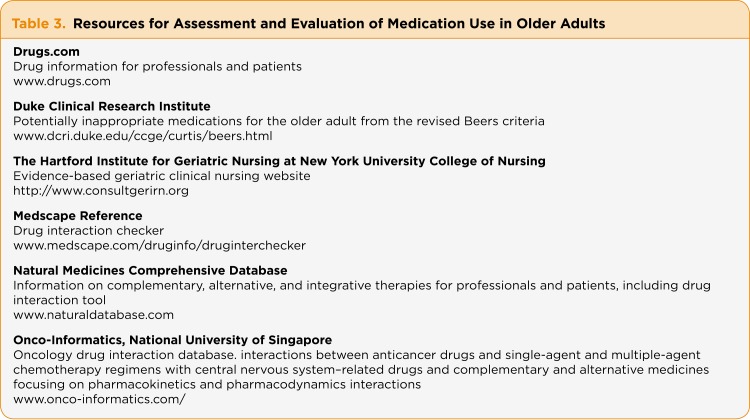
Table 3. Resources for Assessment and Evaluation of Medication Use in Older Adults

In the oncology setting, the evaluation of medication use in the older adult with cancer is even more critical because of the addition of potentially toxic antineoplastic therapy and supportive care medications. The AP should assume a primary role in medication assessment and make appropriate changes as needed. Medications should be reviewed at each visit, especially noting any adverse side effects that the older adult may be experiencing. Patients should be instructed to bring their complete medication list, or the AP may also utilize the "brown bag method," in which patients are instructed to put all medications that they are taking in a brown bag and bring to each visit. The AP should actively involve the patient and caregiver in the review of medications and give verbal and written instructions to prevent medication errors and promote drug safety in this vulnerable population.

## Summary

Having an awareness and understanding of strategies for managing medication regimens in older adults with cancer has become critical for APs as a result of the aging population and prevalence of comorbidities and multiple medications. Knowledge of polypharmacy; effects of normal aging on pharmacotherapeutics; and thorough, ongoing assessment of medications in the older adult with cancer by the AP will ensure optimal drug therapy and prevent adverse drug reactions.
